# Using large language models to facilitate academic work in the psychological sciences

**DOI:** 10.1007/s12144-025-07438-2

**Published:** 2025-01-28

**Authors:** Aamir Sohail, Lei Zhang

**Affiliations:** 1 Centre for Human Brain Health, School of Psychology, University of Birmingham, Birmingham, B15 2TT UK; 2 Institute for Mental Health, School of Psychology, University of Birmingham, Birmingham, B15 2TT UK; 3 Centre for Integrative Neuroscience and Neurodynamics, University of Reading, Reading, UK; 4 School of Psychology and Clinical Language Sciences, University of Reading, Reading, UK; 5 Centre for Developmental Science, School of Psychology, University of Birmingham, Birmingham, B15 2TT UK

**Keywords:** Large Language Models (LLMs), Academia, Psychology, Education, Human behavior, Teaching

## Abstract

Large Language Models (LLMs) have significantly shaped working practices across a variety of fields including academia. Demonstrating a remarkable versatility, these models can generate responses to prompts with information in the form of text, documents, and images, show ability to summarize documents, perform literature searches, and even more, understand human behavior. However, despite providing many clear benefits, barriers remain toward their integration into academic work. Ethical and practical concerns regarding their suitability for various tasks further complicate their appropriate use. Here, we summarize recent advances assessing the capacity of LLMs for different components of academic research and teaching, focusing on three key areas in the psychological sciences: education and assessment, academic writing, and simulating human behavior. We discuss how LLMs can be used to aid each area, describe current challenges and good practices, and propose future directions. In doing so, we aim to increase the awareness and proper use of LLMs in various components of academic work, which will only feature more heavily over time.

## Introduction

Academics are expected to carry out teaching and research duties, having both a commitment to lecturing and grading student work, as well as designing and performing experiments, writing grant and funding applications, and publishing papers. This workload is often excessive, leading to long working hours and feelings of heightened anxiety and inefficiency (Barrett & Barrett, 2008). These burdens may be potentially alleviated by the recently developed large language models (LLMs; Vaswani et al., 2017). LLMs, a specific type of artificial neural networks that are pretrained on statistical relationships in language that ultimately generate a list of outcomes probabilistically representing the most suitable option in response to a given prompt (e.g., “Explain XYZ to first-year undergraduate students), are particularly suitable for specific tasks such as text summarization, knowledge retrieval, and cases where information can be concisely and accurately presented. Subsequently, these models can aid various components of academic work, including in the psychological sciences (Demszky et al., 2023), by summarizing and revising text, analyzing and debugging computer code, performing literature searches and simulating human behaviour.

Teaching and academic writing are activities which particularly stand to benefit from the incorporation of LLMs, given that tasks in the psychological sciences heavily rely on text, verbal or written alike. Academics can use LLMs to freely generate content-relevant material (e.g., numerical cognition in infancy) and automate the grading of assessments; meanwhile, students benefit from LLMs’ utility as a knowledge base and ability to assist learning of practical skills including statistics and programming (e.g., general linear modeling in R). Similarly, LLMs also have significantly altered the writing process for academics, with its ability to propose templated articles, revise and re-word text, and perform literature searches in response to specific queries. However, questions remain regarding their implementation for certain tasks, as LLMs often generate false information in response to specific prompts (Zhang et al., 2023) and false references when performing literature searches (Agrawal et al., 2024). Furthermore, students and academics, while benefitting from increased productivity, conversely face issues relating to plagiarism (Hutson, 2024), critical thinking (Messeri & Crockett, 2024), and hinderances to the learning process (Yan et al., 2023).

Inherently rooted in the psychological sciences (particularly cognitive psychology), a common benchmark for understanding the capability of LLMs involves measuring the response to cognitive tasks and logic puzzles requiring “human-like” reasoning. Early success in this domain prompted research towards using LLMs as proxies for human participants in behavioral experiments, potentially offering the ability to perform complex cognitive tasks more quickly, reliably and cheaply. Responding to behavioral tasks and other assessments submitted as prompts, LLMs are found to replicate classic economic, psycholinguistic, and social psychology experiments (Aher et al., 2023), ultimately demonstrating similarities with human cognition and behavior (Huijzer & Hill, 2023). However, others have noted the various biases inherent with LLMs, including differences between other measures of human decision-making and inference (Crockett & Messeri, 2023), and the inability to reflect more current or constantly changing societal views (Harding et al., 2023). It therefore currently remains unclear for academics in the psychological sciences to which extent LLMs can accurately represent human cognition, and the circumstances where they can accurately provide a substitution for human participants.

Despite the growing body of research, there ultimately remains a significant gap in understanding the nuanced differences in LLM performance across different academic disciplines, particularly in the psychological sciences. Furthermore, the long-term implications of integrating LLMs into academic work have not been thoroughly explored. This article aims to address these gaps by examining how LLMs can effectively enhance academic tasks while proposing strategies to ensure their responsible use. We then discuss the ethical considerations they present and suggest future directions in this rapidly evolving field.

## Large language models in academic education

**Fig. 1:**
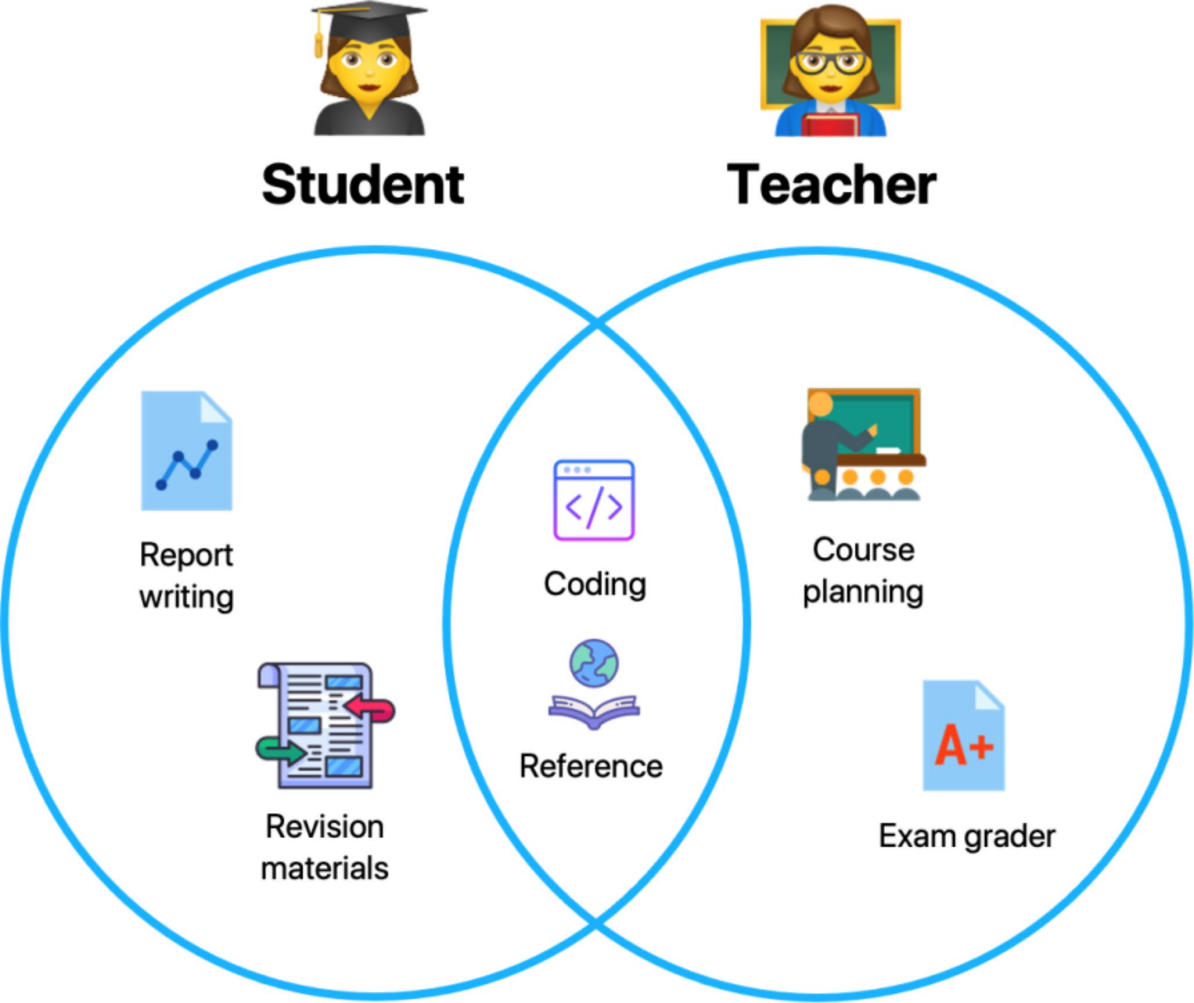
How academics and students can benefit from Large Language Models (LLMs) in higher education. *Note.* Demonstrating their versatility, LLMs offer various benefits for both academics and students, most commonly by providing a knowledge base for key theories and concepts, and as a programming assistant. For students, LLMs can also assist with the revision process and at various stages of written coursework. Teachers can additionally benefit by using LLMs to plan courses and as an exam grader. Icons by Icons8.com

Psychology and related courses within higher education involve both theoretical and practical learning. Academics conceive and deliver concepts, theories, and empirical evidence for key topics in psychology, whereas students are expected to learn and portray critical insight towards those theories, and develop practical skills including statistics, experimental design, and programming. The underlying structure of LLMs make them highly suitable for aiding both theoretical and practical modes of learning, offering a clear benefit to both academics and students alike (Fig. 1). While the benefit for students is more apparent, teaching, at and above the undergraduate level, covers extensive amounts of conceptual information. As certain topics may initially be unfamiliar to the lecturer who will need to refresh their own subject knowledge, LLMs summarize complex topics at an appropriate level relevant for their teaching. LLMs can also be used to plan entire modules and how the content is delivered by creating quizzes and assessments that test students’ understanding of the material throughout the entire semester. This includes generating specific learning materials for those with learning difficulties (e.g., creating Concept Maps from conversations for dyslexic students) (D’Urso & Sciarrone, 2024) and translating materials into different languages for those whose primary language is not English (Lo, 2023). These elements are getting increasingly important considering equality, diversity, and inclusion (EDI) in higher education. Ultimately, LLMs employed through chatbots such as ChatGPT benefit the teaching and learning process for both students and academics, improving student performance, motivation, organization and time management, and promote a more effective and collaborative learning environment (Yan et al., 2023).

From the students’ perspective, LLMs can further benefit learning by generating educational materials such as reading comprehension tasks, interactive code explanations and assessment questions, and by improving student-based feedback of another’s work. However, whether LLMs generally lead to an improvement in academic performance cannot be definitively stated, as there currently is a lack of empirically designed studies, particularly within the context of higher education. The extent to which LLMs can bolster education is also dependent on the user’s technical ability and personal attitudes. For example, certain academics report being reluctant to include LLMs as part of their curriculum due to unfamiliarity and confusion (Zhou et al., 2024). Conversely, many students also do not employ LLMs in their own learning, and if so, are not fully aware of its subtle nuances. Students new to programming – a common scenario in the psychological sciences (to program experiments and perform data analysis) - while aware that ChatGPT and other LLM-chatbots can be used to generate and debug code provided as prompts, may be under-educated in prompt engineering, the specific construction of prompts to receive a more suitable response (Lin, 2024). This is an important skill, as ChatGPT tends to be less capable in providing responses to programming questions if not well prompted (Kabir et al., 2023).

However, some have argued that an over-reliance on LLMs will have a negative influence on the skills and working practices accrued by students. Indeed, when using LLM tools to complete a programming project, students demonstrate practical progress but report hindered learning (Tanay et al., 2024), and an increased reliance on LLMs for programming tasks subsequently lowers performance on critical thinking assessments (Jošt et al., 2024). By over-relying on the LLM to provide the solution, students may not think practically about the specific components of the code, resorting to simply copying and pasting generated code *ad nauseum*. We therefore suggest that students use LLMs in programming tasks (and similar tasks) in a scaffolding fashion – utilizing structures and pointers generated by LLMs as an “extra brain” yet independently evaluating and internalizing the actual solution.

Yet, the lines regarding the appropriate use of LLMs in certain areas of education remain blurred. For example, in a programming class, should students be allowed to use code directly generated by an LLM? As employees are not restricted in the materials and resources available in their profession, some argue that universities should instead embrace LLMs and assess the efficacy in which students can use them to retrieve information and generate solutions. Fully educating students on when (and when not) to use LLMs as part of their degree should therefore constitute a critical part of university-level education, avoiding the potential for an “unfair academic playing field”, created by students unaware of the full capabilities of AI tools, or those who choose not to use it due to ethical considerations. In fact, a substantial number of universities worldwide have published student guidelines and guidance on using LLMs and generative artificial intelligence tools^1^. Meanwhile, online tools and platforms are publicly available (e.g., ChatGPT Detector, GPTZero) to detect work generated by LLMs to avoid overuse and misuse of LLMs in higher education.

Understanding the capabilities of LLMs also allows for academics, lecturers, and module convenors to set the appropriate examinations and assessments for their class. As these aim to measure subject knowledge, practical skills and critical thinking, abilities which can be replicated by LLMs to a degree, certain assessments in the psychological sciences may also need to be adjusted. Attempts to prevent the use of LLMs for aiding assessments include employing AI-detectors for essays, reverting to oral presentations and person written examinations. However, with the proven benefit in improving the learning process for certain areas, academics should remain open with students using LLMs in specific cases where the benefits in productivity can, but do not necessarily lead to, reduced learning. We ultimately advocate that academics are educated, well informed and develop a clear agenda before employing LLMs as a practical tool in their teaching.

## Using large language models to aid academic writing

One of the more controversial issues regarding the use of LLMs within academia is their role with aiding the writing process. As LLMs can summarize, generate, and re-phrase text, journals have been timely to demonstrate their position on the matter, with some disallowing any LLM-generated text, and others requiring clear guidance as to which components of the research paper were influenced or generated. Discerning to which extent LLMs should be used presents a difficult situation. Most would agree that entire paragraphs should not be written, re-written or paraphrased by LLMs; however, if, hypothetically, a human writer re-phrased a paragraph of academic text that coincidentally matched word-for-word an LLM-rephrased paragraph of the same text, should neither be used? Ethical dilemmas also exist on a smaller scale as the writing process naturally involves the repetition of others’ work (this is particularly true for Methods sections in journal articles). Given that summarizing the key results of a paper in a sentence or two can only contain a specific set of words, should LLMs be used to re-format a single sentence to avoid plagiarism?

Large language models are often used to generate text intended for a research article or review paper from scratch by providing descriptions of scientific principles or an overview of a research topic. However, the underlying architecture of LLMs cautions against both uses. Answers provided by LLMs in response to open scientific questions can often be incorrect, or irrelevant, necessitating factual checking from the human user. Furthermore, using LLMs to summarize research areas sometimes generates inaccuracies compared to the published original work (Semrl et al., 2023). Paradoxically, the same study also demonstrated an ability to generate conclusions from provided abstracts indistinguishable from human-generated summaries, demonstrating its suitability for specific uses. More recently, the performance of LLMs towards summarizing literature has improved due to the development of advanced models with larger training sets. Search engines primarily implementing GPT-4 (e.g., SciSpace) can highlight relevant papers with fewer hallucinations and false references than earlier models. While promising, these tools are still in their infancy and face several challenges, including hallucination and relevancy of papers to the prompt. Ultimately, models trained upon enormous volumes of data are still commonly not able to provide the domain-specific accuracy and precision in the information retrieved often essential for literature reviews (Susnjak et al., 2024). One strategy aiming to improve accuracy restricts LLMs to aiding specific components of the literature review. For example, ChatGPT is able to generate research questions, suggest research terms and performs well in filtering and categorizing articles, rivalling human performance for certain review tasks including title/abstract screening, full-text review and data extraction (Khraisha et al., 2024). A two-stage hybrid model where LLMs identify relevant papers and themes, for the subsequent human-centred screening of relevant material presents one such approach (Ye et al., 2024), reducing errors and improving the accuracy of the literature review compared to a human-only workflow. Similar hybrid frameworks have been proposed for identifying elements in empirical papers, where LLMs present a time- and cost-effective approach while maintaining the accuracy observed in human reviewers (Uittenhove et al., 2024).

Using LLMs for proof-reading, editing, and shortening original text generated by the user are generally less contested within academia, as this occurs at the end of the creative process and leads to only minor changes from the original text. Some have likened this particular use of LLMs akin to asking a friend or colleague to proof-read a writing sample, which is unlikely to raise ethical concerns such as plagiarism that may arise under text summarization and generation. While early models were only able to process prompts in the form of text, more recently developed models can process entire documents, providing feedback on manuscripts within the order of seconds. However, base models such as GPT-4 have been criticized for producing generic, non-meaningful comments, leading for tailored frameworks to be developed. Such frameworks typically levy multiple LLMs, assigning each LLM a specific task ultimately providing more meaningful and specific comments than the conventional single-model approach (D’Arcy et al., 2024). In any case, the accessibility of proof-reading and editing services through LLMs can additionally provide high-quality English language to non-native speakers and early-career researchers who would otherwise be placed at a disadvantage when submitting publications. Proof-reading in the academic sphere can also be implemented to facilitate grant writing and to aid peer review, allowing academics to focus more on new research. Of note, journals and research funders do require the explicit declaration of the use of LLMs in the writing process.

LLMs, while able to summarize and generate text as part of the academic writing process, currently demonstrate limitations in accuracy and legitimacy in certain domains, more strongly benefitting understanding and text analysis tasks than literature review tasks. Therefore, despite rapidly generating a rapid, general overview of a subject, they currently fall short of being able to generate a literature review of the standards required in academia (Zimmermann et al., 2024). Assigning certain components of the workflow (e.g., identifying relevant papers) to LLMs can therefore present a more time-effective approach while maintaining accuracy. As with other uses, benchmarking performance specific to searching and summarizing scientific literature is key for identifying their strengths and limitations within this space and supports the ongoing development of LLM workflows in scientific literature analysis.

## Simulating human participants with large language models

Multiple fields of research including psychology, sociology, economics, and neuroscience utilize experiments to assess behaviors as part of their research methodology repertoire. However, despite its importance and usefulness, this process has several challenges and potential limitations, including high financial costs and data quality concerns. Furthermore, human participants testing is also slowed by usually time-consuming ethical and practical components of the research process, requiring informed consent from participants, ethical approval, and additional requirements necessary for studying vulnerable groups. Some of the limitations and challenges associated with running behavioral experiments may therefore be avoided by employing artificial agents, with LLMs substituting for human participants.

One of the original motivations of developing LLMs and/or generative AI was to develop machines that could “think like humans” (Lake et al., 2017). The capacity of LLMs to do so stems from the numerous computational properties that allow these models to mimic and imitate human reasoning and inference (Aher et al., 2023). Certain models are further able to exhibit complex behavior consistent with mentalistic inference (Strachan et al., 2024) and demonstrate similar heuristics and context-sensitive responses akin to loss aversion and effort reduction commonly observed in humans (Suri et al., 2024). LLMs are also more likely to succeed in some tasks and fail other tasks, just as human participants do (Dasgupta et al., 2023), leading for some researchers to state that the particular model tested could pass as a valid subject for some experiments that have been administered (Binz & Schulz, 2023b). The appropriability for LLMs to do so is also improving over time, as important differences with human-like reasoning prevalent in older models disappear almost entirely in more recent ones (Yax et al., 2024), demonstrating the importance of model size and complexity that could match the richness of human behaviors. Certain LLMs also demonstrate zero-shot learning (or generalization), the ability to infer on data that the model have never seen in training, by accurately simulating human responses towards previously unseen cognitive tasks (Binz & Schulz, 2023a). Future research may seek to train LLMs on additional tasks, and novel tasks may eventually be tested on simulated cohorts, reducing time and financial costs in developing behavioral studies.

LLMs can also be experimentally induced into specific behavioral states through prompt engineering. For example, prompting LLMs with positive or negative components (e.g., adding the suffix “this is very important to my career” or “Perhaps this task is just beyond your skill set”) has been found to affect the response generated (Li et al., 2023; Wang et al., 2024). This approach has subsequently been applied to understand psychopathology by inducing behavioral states observed among human cohorts with mental health conditions. By experimentally manipulating the level of “anxiety” through anxiety-inducing and happiness-inducing scenarios, GPT-3.5 recreates performance characteristics observed in humans with high anxiety during a simple multi-armed bandit task, engaging in less exploitation and more exploration, and ultimately leading to worse behavior (Coda-Forno et al., 2023). This and similar results have far-reaching implications for validating diagnostic measures and determining the efficacy of cognitive therapies, potentially in combination with computational and neuroimaging data of mental health conditions (Sohail & Zhang, 2024). Indeed, mindful-based interventions have been shown to reduce high levels of anxiety experimentally induced through traumatic narratives (Ben-Zion et al., 2024). As engineering positively themed prompts to LLMs shares similarities with delivering cognitive-based therapies in humans, prompts can be firstly fine-tuned in LLMs, with winning prompts subsequently tested in human patients. Early research has implemented such an LLM-informed treatment approach by generating dialogue systems based on Cognitive Behavioral Therapy (CBT) scenarios. Subsequently, patients report improved mood change and empathy to prompts generated by GPT-4, with no improvements to those generated by a dialogue model (Izumi et al., 2024).

Future studies could further utilize the same framework (i.e., first establish protocols in LLMs, then test it in humans) to investigate developmental psychopathology. Large language models display a pattern of increasing cognitive ability and rising language complexity in correspondence with child development, if prompted to do so (Milička et al., 2024). However, in the same study, task type, prompt type, and the choice of language model were all found to influence developmental patterns, demonstrating variability with this approach. While LLMs offer a novel framework towards understanding human development, cognitive processes arising during childhood such as conceptual abstraction should ideally be assessed using different tasks and at multiple time points. Altogether, this recent and exciting field reflects similarities in computation between humans and machines, with the potential for a computational psychiatric approach, informed by large language models.

**Fig. 2:**
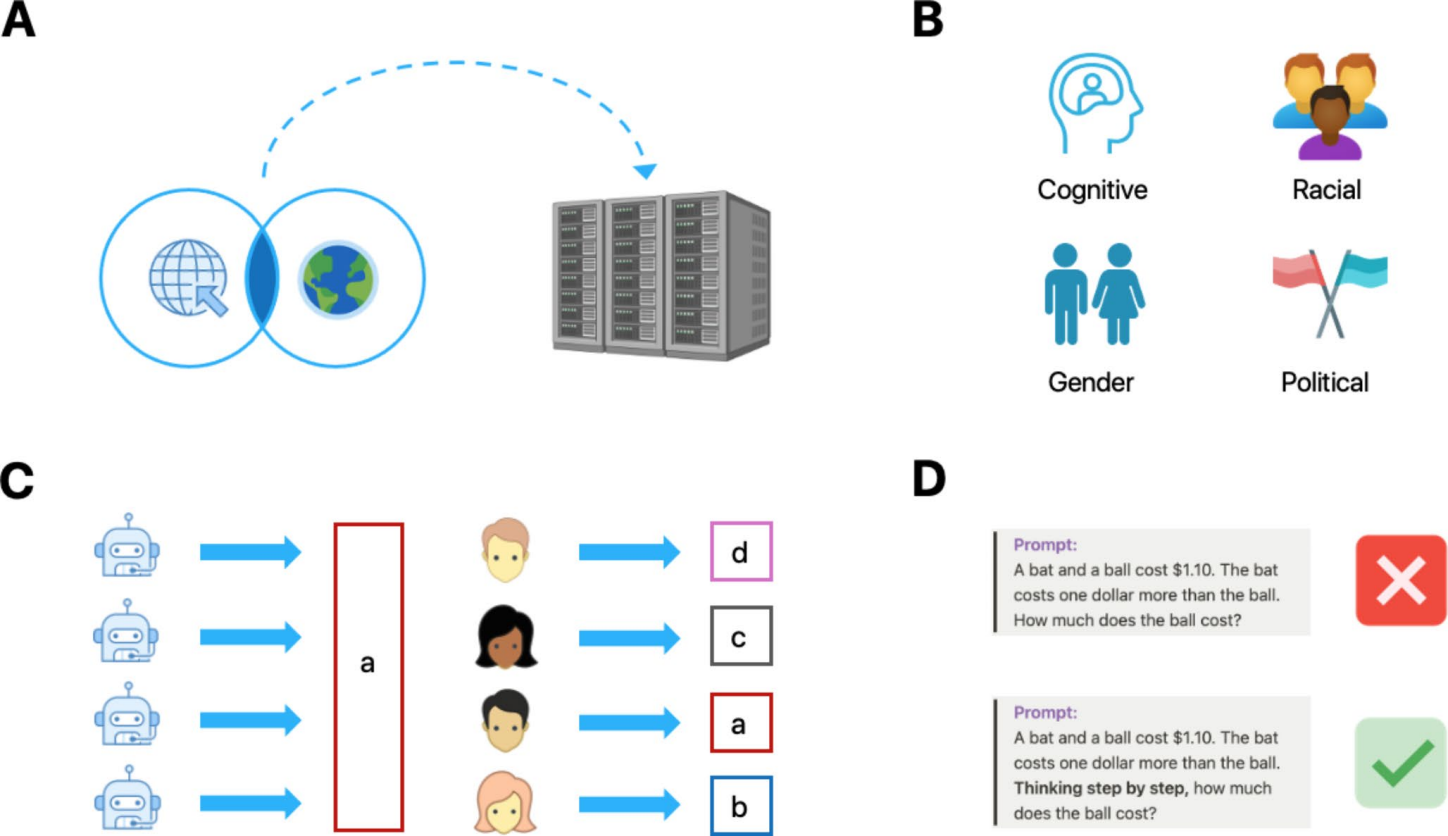
Considerations of employing Large Language Models (LLMs) as proxies for human participants. *Note.* (**A**) Models are trained upon large quantities of online data influenced by those with access to the internet, unrepresentative of the human population. (**B**) LLMs demonstrate several biases including cognitive, racial, gender and political inclinations in their responses to specific prompts. (**C**) In response to questions probing political orientation, economic preference, and moral philosophy, human cohorts demonstrate considerable response variability whereas LLMs demonstrate near-zero variation, a phenomenon dubbed the “correct answer effect”. (**D**) Prompt engineering substantially influences the response provided by LLMs, while having little effect on human-based reasoning. Depicted is the “Chain-of-Thought” (CoT) prompt engineering strategy which improves LLM-based reasoning by breaking down the response into discrete steps. Icons by Icons8.com

While LLMs can - in principle – be used as proxies for human participants, some have advised that this should only be done “when studying specific topics, when using specific tasks, at specific research stages, and when simulating specific samples” (Dillion et al., 2023), reflecting the differences in cognition and behavior observed between humans and machines (Fig. 2). Large language models have been shown to perform differently to human participants in many cognitive tasks, such as those necessitating directed exploration and causal reasoning (Binz & Schulz, 2023b), and during finitely-repeated economic games (Akata et al., 2023). Indicative of a fundamental difference between human and machine thinking, the “correct answer” effect, where questions probing political orientation, economic preference, judgement, and moral philosophy are answered with zero or near-zero variation (Park et al., 2023), rules out the substitution of LLMs as human participants for certain tasks. There are also questions into whether LLMs should even be used at all in this manner, as the training sets of LLMs are overinfluenced by those from Western, Educated, Industrialized, Rich, Democratic (WEIRD) countries as well as those with attitudes that are Hegemonic, Young, and Publicly ExpRessed (HYPER-WEIRD; Crockett & Messeri, 2023). Consequently, these models may lack sufficient diversity in their responses to accurately represent a representative population sample (Wang et al., 2024). ChatGPT, for example, demonstrates gender (Ghosh & Caliskan, 2023), cultural (Cao et al., 2023), and political (Hartmann et al., 2023) biases in its responses, and shows significantly less variance compared to human participants across a range of self-report measures spanning various psychological domains, such as personality, cognition, political orientation, and emotions (Atari et al., 2023). Substituting participants for LLMs could therefore propagate the over-sampling of a specific sub-population, the antithesis of psychological research which is often to obtain samples from and to make inferences towards diverse populations.

Despite these concerns, LLMs provide a tangible benefit as proxies for human participants for specific experimental designs not susceptible to cognitive or variational biases. Looking forward, this promising field should further identify the similarities and differences between LLMs and human behavior by developing testable and ethologically meaningful benchmarks, frameworks guiding experimenters whether to integrate LLM-generated data into their research pipeline, and prompt datasets for mitigating against cognitive biases. Furthermore, making publicly available articles, tutorials, and notebooks detailing how the lay-psychology researcher can substitute LLMs for human participants (Hussain et al., 2023) will make this often technically difficult research more accessible within the psychological sciences.

## Conclusion

LLMs contest a highly debated area of academic research including the psychological sciences. Not quite the “academic panacea” some have made it to be, LLMs nevertheless constitute an integral part of the academic workflow for an increasing number. Academics currently use LLMs to write essays and talks, summarize literature, draft and improve papers, identify research gaps, write computer code and perform statistical analyses. As time progresses, this capability will only increase, evolving to the point that LLMs are expected to design experiments, write and complete manuscripts, conduct peer review and support editorial decisions to accept or reject manuscripts.

Furthermore, domain-specific LLMs stand to increase academic performance and productivity within specific fields. Within psychology, this is particularly promising for simulating human behavior and cognition, allowing researchers to test cognitive models and theories with greater precision and efficiency. While careful consideration must be given to their proper use, we advocate for LLMs to be openly endorsed by academics in psychology and beyond.

## Footnotes

1 One of the example guidance is from the authors’ affiliation: University of Birmingham (UK)’s Student guidance on using Generative Artificial Intelligence tools ethically for study. [retrieved on 09 July 2024]. https://intranet.birmingham.ac.uk/as/libraryservices/asc/student-guidance-gai.aspx.
